# Prevalence of lifestyle cardiovascular risk factors and estimated framingham 10-year risk scores of adults with psychotic disorders compared to controls at a referral hospital in Eldoret, Kenya

**DOI:** 10.1186/s12888-023-05409-0

**Published:** 2023-12-05

**Authors:** Edith Kwobah, Nastassja Koen, Ann Mwangi, Lukoye Atwoli, Dan J. Stein

**Affiliations:** 1 Department of Psychiatry, Moi Teaching and Referral Hospital, Eldoret, Kenya; 2 Department of Psychiatry and Mental Health & Neuroscience Institute, South African Medical Research Council (SAMRC) Unit on Risk and Resilience in Mental Disorders, University of Cape Town, Cape Town, South Africa; 3 Department of Mathematics, Physics and Computing, School of Science and Aerospace Studies, Moi University, Eldoret, Eldoret, Kenya; 4 Brain and Mind Institute, Department of Medicine, The Aga Khan University, East Africa, Nairobi, Kenya; 5 South Africa Medical Research (SAMRC) Unit on Risk & Resilience in Mental Disorders, Department of Psychiatry and Neuroscience Institute, University of Cape Town, Cape Town, South Africa

**Keywords:** Lifestyle, Cardiovascular risk, Psychotic disorders, Eldoret, Kenya, Risk score

## Abstract

**Introduction:**

Lifestyle factors such as smoking, alcohol use, suboptimal diet, and inadequate physical activity have been associated with increased risk of cardiovascular diseases. There are limited data on these risk factors among patients with psychosis in low- and middle-income countries.

**Objectives:**

This study aimed to establish the prevalence of lifestyle cardiovascular risk factors, and the 10-year cardiovascular risk scores and associated factors in patients with psychosis compared to controls at Moi Teaching and Referral Hospital in Eldoret, Kenya.

**Methods:**

A sample of 297 patients with schizophrenia, schizoaffective disorder, or bipolar mood disorder; and 300 controls matched for age and sex were included in this analysis. A study specific researcher-administered questionnaire was used to collect data on demographics, antipsychotic medication use, smoking, alcohol intake, diet, and physical activity. Weight, height, abdominal circumference, and blood pressure were also collected to calculate the Framingham 10-year Cardiovascular Risk Score (FRS), while blood was drawn for measurement of glucose level and lipid profile. Pearson’s chi-squared tests and t-tests were employed to assess differences in cardiovascular risk profiles between patients and controls, and a linear regression model was used to determine predictors of 10-year cardiovascular risk in patients.

**Results:**

Compared to controls, patients with psychosis were more likely to have smoked in their lifetimes (9.9% vs. 3.3%, p = 0.006) or to be current smokers (13.8% vs. 7%, p = 0.001). Over 97% of patients with psychosis consumed fewer than five servings of fruits and vegetables per week; 78% engaged in fewer than three days of vigorous exercise per week; and 48% sat for more than three hours daily. The estimated 10-year risk of CVD was relatively low in this study: the FRS in patients was 3.16, compared to 2.93 in controls. The estimated 10-year cardiovascular risk in patients was significantly associated with female sex (p = 0.007), older patients (p < 0.001), current tobacco smoking (p < 0.001), and metabolic syndrome (p < 0.001).

**Conclusion:**

In the setting of Eldoret, there is suboptimal physical exercise and intake of healthy diet among patients with psychosis and controls. While the estimated risk score among patients is relatively low in our study, these data may be useful for informing future studies geared towards informing interventions to promote healthy lifestyles in this population.

## Background

Patients with mental illness have an increased mortality rate compared to the general population [1]. Cardiovascular disorders (CVDs), the leading cause of death worldwide [2], contribute significantly to the excess mortality among patients with mental illness, with CVDS accounting for 17.4% and 22.0% of the reduction in life expectancy in males and females, respectively [3]. While early detection and management of these patients have potential to improve outcomes, sadly people with mental disorders receive less screening and lower-quality treatment for CVD. Modifiable CVD risk factors include smoking, alcohol intake, physical inactivity and unhealthy diet [4].

Smoking is one of the leading causes of premature deaths globally and is among the five leading risk factors for ill health, as measured by disability-adjusted life-years (DALYS) [5]. Smoking may lead to accelerated atherogenesis, increased risk of ischemia, arrhythmias and myocardial infarction [6]. The mechanisms underlying increased tobacco use in the mentally ill are likely bidirectional - smoking may increase the risk of psychosis; and patients with psychosis may smoke to alleviate the negative experiences resulting from their symptoms [7].

Harmful alcohol use is the seventh leading cause of death worldwide and a major contributor to DALYS [8]. Excess alcohol intake increases the risk of CVD by contributing to atherosclerosis, increasing rates of hypertension, arrhythmias and dilated cardiomyopathy [9] The pathways underlying comorbid alcohol use disorder and psychotic disorders are likely multifaceted. For example, patients with schizophrenia may overvalue drug-like reward experiences in addition to experiencing greater euphoria and stimulatory effects in response to alcohol); and may devalue the potential negative consequences of substances. This may be further complicated by detrimental decision-making and impulsive behaviour in this patient group [10].

Inadequate physical activity and sedentary behaviour are both common contributors to many chronic diseases; [11] and are classified as modifiable CVD risk factors [12, 13]. Adequate physical activity is a cost-effective way of promoting cardiovascular health [14]. Metanalytic evidence indicates that an increase from inactivity to achieving 2.5 h of moderate-intensity aerobic activity/ week, is associated with lower risk of CVD mortality [15–17].

The benefits of adequate fruit and vegetable intake (i.e. at least half a plate/day) in mitigating CVD risk factors such as DM and obesity, are well documented [18, 19]. Existing literature indicates that about 200 g of fruits and vegetables consumed per day significantly reduces the relative risk for coronary heart disease and for all-cause mortality 0.90 [20]. Studies assessing the dietary patterns of patients with schizophrenia report that these individuals often follow a poor diet (e.g. with increased intake of saturated fats and reduced intake of fruits and vegetables), thus augmenting the risk of metabolic disturbances [21]. Poor dietary habits among patients with psychosis have also been linked to unemployment, negative symptoms of psychosis, economic challenges and low knowledge levels on importance of a healthy diet [22].

To quantify risk and guide intervention, scores have been developed to estimate the 10- year risk of developing CVD. Of these scores, the Framingham risk score (FRS) is the most commonly used in patients with mental illness [23]. FRS is a multivariable risk factor algorithm used to assess general CVD risk and risk of individual CVD events, including coronary, cerebrovascular, and peripheral arterial disease and heart failure [24] The scores are categorized as low (< 10), intermediate [10–19] and high > 20. FRS has been reported to be significantly higher among patients with psychosis compared to the general population [25].

While the burden and sequelae of CVDs may be reduced through simple and cost-effective methods such as smoking cessation, improved diet and physical activity, as well as substance use interventions; there is limited evidence from low- and middle-income countries (LMICs) and from Sub-Saharan Africa (SSA) on the prevalence of these risk factors, and on how they relate to overall cardiovascular risk profile. In addition, minimal literature has documented the estimated 10-year cardiovascular risk scores in these settings. This dearth of evidence has likely contributed to limited clinical programs addressing CVD risk factors in patients with psychosis in LMICs and SSA. Thus, this study had two objectives;

1. To determine the prevalence of modifiable lifestyle CVD risk factors among patients treated for psychotic disorders compared to controls at Moi Teaching and Referral Hospital (MTRH) in Eldoret.
2. To document the estimated 10-year risk score and associated factors among patients with psychosis compared to controls in this setting.

## Materials and methods

This was an observational study conducted in 2019 at Moi Teaching and Referral Hospital (MTRH), situated in western Kenya, as part of PhD work nested within the larger Neuropsychiatric Genetics of African Populations (NeuroGAP) initiative [26].

For this study, convenience sampling was done. A sample size calculation was carried out as recommended by Peduzzi et al. (1996).These authors recommended that the minimum number of participants to include in a study such as ours should be obtained as follows: N = 10 k/p where K is the number of covariates and p is the proportion of cases in the population [27]. Using an estimated prevalence of MetS in Kenya of approximately 35% [28] and 10 covariates in the data collection tool: N = 10 × 10/0.35 = 285.7. This sample size is comparable to one prior study by Saloojee et al. which employed a design similar to ours and was conducted in South Africa [29]. To allow for missing data and quality control procedures, we recruited 300 patients and 300 controls 300 controls frequently matched for age and sex.

Patients were recruited from the booked outpatient consultant clinic at MTRH that takes place every Wednesday of the year. According to the hospital records about 5000 patients are seen in the clinic every year and 60% of these have psychotic disorders. For the purposes of this study, “apsychotic disorder” was defined as schizophrenia, schizoaffective and bipolar mood disorders as captured in the *Diagnostic and Statistical Manual, fifth edition* (DSM-5) [30]. On the clinic date participants with a documented diagnosis of bipolar mood disorder, schizoaffective disorder and schizophrenia were identified from the waiting bay using their name and the personal file number. They were given more information on the study and requested to participate.

Adult participants with schizophrenia, schizoaffective disorder, or bipolar mood disorder, with ability to consent were eligible for inclusion. We excluded pregnant women and those who did not have capacity to consent due to cognitive impairment as defined by a score less than 14.5 on the University of California, San Diego Brief Assessment of Capacity to Consent (UBACC). Capacity to consent was defined by a score of at least 14.5 and above on the University of California, San Diego Brief Assessment of Capacity to Consent (UBACC). UBACC is a 10-item scale with good internal consistency, interrater reliability and concurrent validity [31]. It also has a short administration time and simple language making it easy to administer. It was translated into Swahili for use in this study setting. The tool was administered in Swahili or English based on the participants preference. Participants with a score less than 14.5 after a maximum of four trials, indicating cognitive inability to consent, were excluded from the study.

Recruitment of control participants took place every day of the week except Wednesday- Wednesday was reserved for patient recruitment given that the clinic only runs once a week. Posters were displayed on the hospital notice boards informing members of the public of the study- students and persons visiting the hospital. Those who expressed interest to participate in the study were asked to contact the research assistant for further information, either telephonically or in person at the hospital. Those who approached the research assistant expressing interest to participate were given more details about the study, and upon accepting to participate written informed consent was taken.

For the control group we included adults aged 18 years and above who consented to participate in the study. We excluded persons known to have an existing mental illness or who were taking any psychiatric medication as determined by self-report or medical record review. We also excluded persons receiving medical treatment for acute alcohol or drug intoxication, as well as pregnant women. Pregnant women were excluded due to the evidence that substantial changes occur in pregnancy including insulin resistance, raised serum glucose as well as changes in lipid metabolism [32].

A researcher-administered questionnaire was used to collect data on demographic variables including age, sex, education level, marital status, current medication use (including antipsychotic medication) and smoking history. The Alcohol use Identification Test (AUDIT – C) was used to screen for harmful alcohol use [33]. The AUDIT-C comprises three questions, each scored on a 5-point Likert scale (minimum score = 0; maximum score = 4), with the total score ranging from 0 to 12. A score of ≥ 4 in men and ≥ 3 in women has been used previously for identifying an alcohol use disorder [33, 34].

Items assessing diet and exercise were adapted from the WHO Steps Survey, a tool used to measure risk factors for non-communicable diseases [35]. In the current study, we asked patients to reflect about a typical week. We classified adequate exercise as 3–7 days a week of either: (1) vigorous intensity activities that increase one’s breathing and heart rate, as part of one’s work; or (2) walking or riding a bicycle for at least 10 min continuously to get to and from places; or (3) doing vigorous-intensity sports, fitness or recreational (leisure) activities. Fewer than three days of these were classified as inadequate. Sedentary behaviour was estimated in terms of amount of time spent sitting or reclining as follows: (1) fewer than 3 h in a day (active) or (2) more than 3 h in a day (sedentary). Intake of fruits and vegetables was quantified as number of servings per week. Five or more servings per week was classified as adequate, while fewer than five were classified as inadequate.

In addition to the researcher-administered questionnaires, measurements for weight, height, abdominal circumference, and blood pressure were taken and blood was drawn for blood sugar and a non-fasting lipid profile. These were used to make a diagnosis of metabolic syndrome which according to Adult Treatment Panel III includes Abdominal obesity > 102 cm in men, > 88 cm in women, Triglycerides ≥ 150 mg/dl, High Density Lipoproteins < 40 mg/dl or 1mmol/l in men, < 50 mg/dl or 1.3mmol/l in women, Blood pressure ≥ 130/≥85mmhg or treatment for hypertension, Fasting glucose- ≥ 6.1 mmol/lor treatment for diabetes, Random Blood sugar ≥ 11.1 mmol/l [36].

### Statistical analyses

Statistical analyses were conducted in Stata version 15 for analysis the frequencies of CVD risk factors (i.e., smoking, alcohol intake, exercise) Pearson’s chi-squared tests were used to determine differences between patients with psychosis versus controls. FRS was computed using participant age, sex, total cholesterol, High Density Lipoprotein cholesterol, smoking habits, and systolic blood pressure. The scores were then categorized as low (< 10), intermediate (10–19) or high (> 20). T-tests were used to determine the differences in the means of the Framingham 10-year estimated risk for cardiovascular disorders between the patients and the controls. Logistic regression modelling was then undertaken to explore the predictors of a high FRS in patients with psychosis.

## Results

A total of 700 participants were approached, and of these 597 participants (297 cases and 300) controls participants were included in this study, the. As shown in Table 1 below, participants in this study sample were young, with a mean age of 33 years among the patients and 35 years among the controls. Cases were more likely to be married compared to controls (35%vs 55%, p < 0.001) Cases were more likely to be unemployed compared to controls (48% vs. 23%, p < 0.001). Cases were less likely to have attained tertiary education (26%vs 56% P < 0.001).

**Table 1:** Sociodemographic characteristics of participants

Variable	Mean (SD) or n (valid%)	Mean (SD) or n (valid%)	Mean (SD) or n (valid%)	T or Χ^2^ (df)	*P-value*
	Total	Case	Control		
**Age in years**	34.81 (10.33)	34.53 (10.44)	35.08 (10.22)	0.64 (595)	0.519^3^
**Sex**				1.39 (1)	0.238^1^
Male	265 (44.4)	139 (46.8)	126 (42.0)		
Female	332 (55.6)	158 (53.2)	174 (58.0)		
**Marital status**				26.06(2)	< 0.001^1^
Married	269 (45.1)	103 (34.7)	166 (55.3)		
Never married	239 (40.0)	139 (46.8)	100 (33.3)		
Widow/separated/divorced	89 (14.9)	55 (18.5)	34 (11.3)		
**Highest level of Education**				67.10(3)	< 0.001^1^
None	35 (5.9)	23 (7.7)	12 (4.0)		
Primary	147 (24.6)	106 (35.7)	41 (13.7)		
Secondary	172 (28.8)	92 (31.0)	80 (26.7)		
Tertiary	243 (40.7)	76 (25.6)	167 (55.7)		
**Occupation**				80.84(2)	< 0.001^1^
Unemployed	217 (36.3)	143 (48.1)	74 (24.7)		
Formal	187 (31.3)	43 (14.5)	144 (48)		
Self	193 (32.3)	11(17.4)	82 (27.3)		

^*1*^ *Chi square test* ^*3*^ *t-test*

Compared to controls, patients were more likely to have smoked in their lifetimes (13.8% patients vs. 7% controls, p = 0.006) or to be current smokers (9.8% vs. 3.3%, p = 0.001). Almost all patients (97%) consumed inadequate fruits and vegetables; most (78%) had inadequate physical activity and almost half (48%) had significant sedentary behaviour. Of note, statistically significant differences in physical activity and dietary habits were not found between patients and controls, Table 2.

**Table 2:** Lifestyle CVD risk factors

	Total	Cases N (297)	Controls N (300)	Χ^2^	*P-value*
Variable	n (valid %)	n (valid%)	n (valid %)		
**Current smoker**				**10.11(1)**	**0.001** ^**1**^
No	558 (93.5)	268 (90.2)	290 (96.7)		
Yes	39 (6.5)	29 (9.8)	10 (3.3)		
**Ever smoked**				**7.42(1)**	**0.006** ^**1**^
No	535 (89.6)	256 (86.2)	279 (93.0)		
Yes	62 (10.4)	41 (13.8)	21 (7.0)		
**Harmful alcohol**				0.21(1)	0.648^2^
No	579 (97.0)	289 (97.3)	290 (96.7)		
Yes	18 (3.0)	8 (2.7)	10 (3.3)		
**Poor diet**				0.87(1)	0.352^2^
No	11 (1.8)	7 (2.4)	4 (1.3)		
Yes	586 (98.2)	290 (97.6)	296 (98.7)		
**Inadequate exercise**				2.98(1)	0.084^1^
No	114 (19.1)	65 (21.9)	49 (16.3)		
Yes	483 (80.9)	232 (78.1)	251 (83.7)		
**Time spent sitting**				0.01(1)	0.906^1^
< 3 hours in a day	311 (52.1)	154 (51.9)	157 (52.3)		
> 3 hours in a day	286 (47.9)	143 (48.1)	143 (47.7)		

^*1*^ *Chi square test* ^*2*^ *Fishers’ exact test*

### 10-year estimated risk of developing CVDs

In this study, the FRS among patients with psychosis was 3.16, which was comparable to that in the control group (2.93). Table 3. Most patients (94.6%) had a low-risk score (< 10), 4.4% had an intermediate risk and 1.0% had high risk for CVD.

**Table 3:** A comparison of Framingham Risk Score between patients and controls

	Total n (valid%)	Control n(valid%)	Case n(valid%)	χ^2^	*P-value*
**Framingham Score**				0.02(2)	1.000
Low risk	563 (94.3)	282 (94.0)	281 (94.6)		
Intermediate	27 (4.5)	14 (4.7)	13 (4.4)		
High Risk	7 (1.2)	4 (1.3)	3 (1.0)		

**Table 4:** Linear regression model with 10-year Framingham Risk Score as the outcome among patients

	10-year Framingham Risk score			
	Coefficient	SE	T	*P-value*
Female	-1.13	0.35	-3.25	**0.001**
Age	0.24	0.02	14.73	**< 0.001**
**Employment**				
Formal vs None	-0.52	0.48	-1.09	0.278
Self-employment vs None	-0.58	0.36	-1.61	0.109
**Education**				
Primary vs None	-0.26	0.62	-0.42	0.673
Secondary vs None	-0.34	0.63	-0.54	0.590
Tertiary vs None	-0.00	0.65	-0.00	0.997
Obesity	0.49	0.34	1.42	0.156
Current tobacco use	3.75	0.57	6.62	**< 0.001**
Harmful alcohol use	1.02	1.00	1.02	0.308
Metabolic syndrome	2.89	0.42	6.88	**< 0.001**
**Olanzapine dose**				
< 10mg vs none^1^	0.49	0.47	1.05	0.295
> 10mg vs none	0.36	0.53	0.67	0.504

^1^Olanzapine was used because 85% of the patients were on olanzapine being a donated drug in this setting

## Discussion

This study describes the modifiable CVD risk factors and estimated 10-year risk score among patients managed for psychotic disorders in Kenya and how this compares to the general population. To the best of our knowledge, this is the first study to explore this in this setting. A key finding was that patients with psychosis (compared to controls) were more likely to have smoked in their lifetimes, or to be current smokers. Most patients consumed inadequate fruits and vegetables, and had low levels of physical activity, almost half of patients reported sedentary behaviour but these findings were comparable to the control group. Of note, the estimated 10-year cardiovascular risk among patients (and controls) was low. In the patient group, this risk was found to be associated with female sex, current tobacco smoking and presence of metabolic syndrome.

In this study, 13.8% of patients reported cigarette smoking. This finding is comparable to the prevalence of smoking (13.5%) in the general population in Kenya [37]. However, a recent study of patients with schizophrenia in Nigeria reported a lifetime prevalence of 25% [38]. Moreover, a review and meta-analysis (including 61 studies, 72 samples and a total of 14,555 tobacco users and 273,162 non-users) reported a smoking prevalence of 57%, amongst patients with first episode psychosis [39]. The lower prevalence found in the current study may be attributable to underreporting of smoking, potentially due to perceived and/or) internalized stigma associated with substance use. The higher prevalence of smoking among patients versus controls in the current study is also in line with well-established evidence. For example, patients with psychotic disorders have been reported to be 4 to 6 times more likely to smoke, compared to those without psychosis [40].

In the current study, only 2.7% of the patients with psychosis reported harmful alcohol use - this did not differ significantly from the control group (3.3%). This prevalence is notably lower than expected, given that a recent community-based study in the same region of Western Kenya found that the prevalence of harmful alcohol use was 12% [41]. Further, one meta-analysis of 123 studies (totalling 165,811 participants) reported that 24% of patients with schizophrenia had a co-morbid alcohol use disorder [42]. This lower prevalence of harmful alcohol use in our study may reflect social desirability bias due to attitudes related to alcohol use in this setting [43, 44], thus suggesting a need for more objective measures of alcohol use such as blood or urine tests in future work.

In this study, almost all patients with psychosis as well as controls reported eating less than one serving of fruit per week, and fewer than three servings of vegetables per week. This trend of sub-optimal intake of fruits and vegetables was also reported in a Kenyan cross sectional study that only 12.4% of participants had two or more servings of fruit a day, only 7.4% had three or more servings of vegetables a day and 94.0% had less than five servings of fruits and vegetables a day [45]. The suboptimal intake of fruits and vegetables has been attributed to symptoms of the illness (particularly negative symptoms and cognitive impairment); as well as to low financial abilities, which would limit purchasing power for healthy meals [46, 47].

This study reported inadequate exercise among a vast majority of patients and controls, as quantified by number of physical activities per week. Though no study has documented physical activity among patients with psychosis in Kenya, the 2015 WHO Steps Survey, reported that up to 80% of the population in Kenya do not engage in adequate physical activity, which could be assumed to include those with psychosis [48]. Similarly, a recent systematic review (including a total of 2,033 patients with psychosis reported that patients with psychosis may spend up to 9 h of their wakefulness in a sedentary state; and at least 2 h more is spent in a sedentary state, compared to the general population [49]. Another systematic review of 25 studies reported an association between low physical activity in patients with psychosis and negative symptoms (e.g. anhedonia); side-effects of antipsychotic medication, lack of knowledge of CVD risk factors, lack of belief in the health benefits of physical activity and lower self‐efficacy; and social isolation [50]. Similarly, a review of 35 studies (totalling 3,453 individuals with schizophrenia found an association between low physical activity levels, and comorbid depressive symptoms [51].

In the current study, almost half of the patients reported spending more than three hours per day sitting, which did not differ significantly from the controls. This is in contrast to findings of a global meta-analysis that patients with mental illness have more sedentary behaviour, when compared to the general population [52]. Further, a systematic review and meta-analysis (including 2,033 participants with psychosis) reported that patients spent an average of 11 h per day in sedentary behaviour – about 2.8 h more than the general population [49]. Contributing factors to low physical activity in this patient population may include impaired cognition, depression, mobility difficulties and self-care challenges.

The estimated 10-year risk of developing cardiovascular-related events among the patients in this study was low. This finding is in line with recent work in Brazil among patients with psychosis which reported that only 1.2% of the study population had severe FRSs [53]. This is in contrast to the findings of the Clinical Trials of Antipsychotic Treatment Effectiveness (CATIE) schizophrenia study which reported significantly elevated FRSs among patients with schizophrenia compared to controls – both for males (9.4% in patients vs. 7.0% in controls) and females (6.3% vs. 4.2%) [54]. Similarly, a study in Lebanon of 329 patients with schizophrenia reported that 31.6% of the participants had intermediate risk of developing CVD, while 7.6% had high risk [55]. Further, a study in China (of 83 patients with schizophrenia and 243 controls) also reported that patients had a higher mean 10-year CVD risk (4.6%), compared to controls (3.1%) [56]. These inconsistencies may – in part – reflect the young age of participants in the current study [57], as well as the overall low CVD risk in the study setting. For example, one cross-sectional survey of slum dwellers in Nairobi found that the CVD risk score was low with only 1.7% of the study sample having a high risk score [58]. In this study, FRSs were significantly associated with female sex and smoking. These findings are consistent with those of a study in China of 83 patients with schizophrenia and 243 controls, which reported that smoking and Metabolic disorder were the leading contributors of increased CVD risk [56].

The current study provides novel and clinically relevant preliminary data of lifestyle CVD risk profile among patients with psychosis in Eldoret, Kenya. Nonetheless, several methodological limitations should be borne in mind when interpreting these study findings. First, an observational study design was undertaken, thus potential causal relationships between psychosis and lifestyle CVD risk could not be ascertained. Second, most data on tobacco use, alcohol consumption, exercise and diet were self-reported, which is likely to have introduced reporting bias. Third, the use of absolute scores to describe CVD risk is limited. For example, the relatively low FRS in our patient group may have been skewed by the young age of the study participants. In addition, FRSs represent average values without considering individual variability in risk. In future, studies assessing the FRS in older participants in our setting; comparing utility of different risk scores would be considered. Lastly, we acknowledge the limitation associated with the enrolment process for cases and control which limits the understanding this may impact on the results.

Despite these limitations, this study highlights the burden of lifestyle risk factors among patients with psychotic disorders in Western Kenya. To address this burden, strategies that are achievable in resource-limited settings are needed. For example, incorporating lifestyle modification activities (with attention to diet and exercise) into routine care would be beneficial in promoting both the mental well-being and cardiovascular health of patients with psychosis [59]. Such activities may include targeted behavioural weight loss [60, 61], in which physicians collaboratively identify physical activity goals for specific patients, reinforce efforts to reach the targets, and continuously address barriers to physical activity [62]. Clinicians should also routinely discuss and encourage healthy and culturally appropriate diets (such as fresh fruit and vegetables and meals made at home); and discourage unhealthy foods (such as fast foods and hotel foods) [63]. Early involvement of nutritionists for individual and group support (where possible) is also key in promoting healthy diets [64]. In addition, strategies should be employed to mitigate potential poor adherence to these lifestyle changes; and to promote long-term reduction in CVD risk [65]. Such strategies may include psychoeducation group sessions and use of incentives [66].

Prevention or cessation of alcohol consumption and cigarette smoking should also be incorporated into the care of patients with psychosis [67]. Although smoking cessation is an efficacious and cost-effective method of improving cardiovascular health [68], cessation therapies are not widely implemented. This may, in part, be due to the perception that smoking is a “lifestyle choice”; and that relapse is common [69]. Medication to assist smoking cessation, such as bupropion and nicotine replacement therapies - as well as group and individual psychotherapies - should also be routinely incorporated into care, where feasible [70]. Further work is recommended to establish the causal and temporal relationships between psychosis and CVD risk factors and how best to promote physical health among patients with psychosis.

## Data Availability

The datasets used and/or analyzed during the current study are available from the corresponding author on reasonable request.

## Abbreviations

CVD: Cardiovascular Disorders
FRS: Framingham risk score
MTRH: Moi Teaching and Referral Hospital
NeuroGAP: Neuropsychiatric Genetics of African Populations
WHO: World Health Organization
