# Correction: Prevalence of lifestyle cardiovascular risk factors and estimated framingham 10-year risk scores of adults with psychotic disorders compared to controls at a referral hospital in Eldoret, Kenya

**DOI:** 10.1186/s12888-024-05551-3

**Published:** 2024-02-09

**Authors:** Edith Kwobah, Nastassja Koen, Ann Mwangi, Lukoye Atwoli, Dan J. Stein

**Affiliations:** 1 Department of Psychiatry, Moi Teaching and Referral Hospital, Eldoret, Kenya; 2 Department of Psychiatry and Mental Health & Neuroscience Institute, South African Medical Research Council (SAMRC) Unit On Risk and Resilience in Mental Disorders, University of Cape Town, Cape Town, South Africa; 3 Department of Mathematics, Physics and Computing, School of Science and Aerospace Studies, Moi University, Eldoret, Eldoret, Kenya; 4 Brain and Mind Institute, Department of Medicine, The Aga Khan University, East Africa, Nairobi, Kenya; 5 South Africa Medical Research (SAMRC) Unit On Risk & Resilience in Mental Disorders, Department of Psychiatry and Neuroscience Institute, University of Cape Town, Cape Town, South Africa

**Correction: BMC Psychiatry 23: 909 (2023)**

**https://doi.org/10.1186/s12888-023-05409-0**

Following the publication of the original article [1], the authors identified errors in Tables 1, 2, 3 and 4. The correct tables are given below.

The incorrect Table 1 is: 
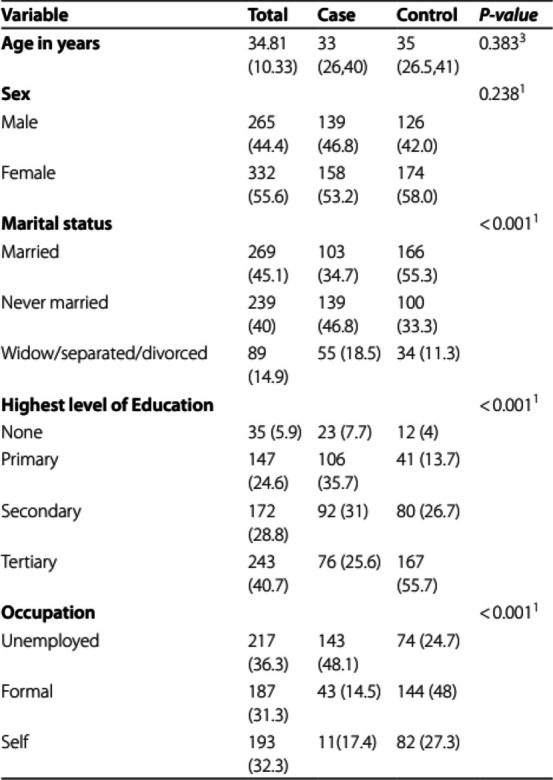


The correct Table 1 is:

**Table 1:** Sociodemographic characteristics of participants

**Variable**	**Mean (SD) or n (valid%)**	**Mean (SD) or n (valid%)**	**Mean (SD) or n (valid%)**	T **or Χ**^**2**^** (df)**	***P*-value**
	**Total**	**Case**	**Control**		
**Age in years**	34.81 (10.33)	34.53 (10.44)	35.08 (10.22)	0.64 (595)	0.519^3^
**Sex**				1.39 (1)	0.238^1^
Male	265 (44.4)	139 (46.8)	126 (42.0)		
Female	332 (55.6)	158 (53.2)	174 (58.0)		
**Marital status**				26.06(2)	< 0.001^1^
Married	269 (45.1)	103 (34.7)	166 (55.3)		
Never married	239 (40.0)	139 (46.8)	100 (33.3)		
Widow/separated/divorced	89 (14.9)	55 (18.5)	34 (11.3)		
**Highest level of Education**				67.10(3)	< 0.001^1^
None	35 (5.9)	23 (7.7)	12 (4.0)		
Primary	147 (24.6)	106 (35.7)	41 (13.7)		
Secondary	172 (28.8)	92 (31.0)	80 (26.7)		
Tertiary	243 (40.7)	76 (25.6)	167 (55.7)		
**Occupation**				80.84(2)	< 0.001^1^
Unemployed	217 (36.3)	143 (48.1)	74 (24.7)		
Formal	187 (31.3)	43 (14.5)	144 (48)		
Self	193 (32.3)	11(17.4)	82 (27.3)		

^1^Chi square test

^3^t-test

The incorrect Table 2 is: 
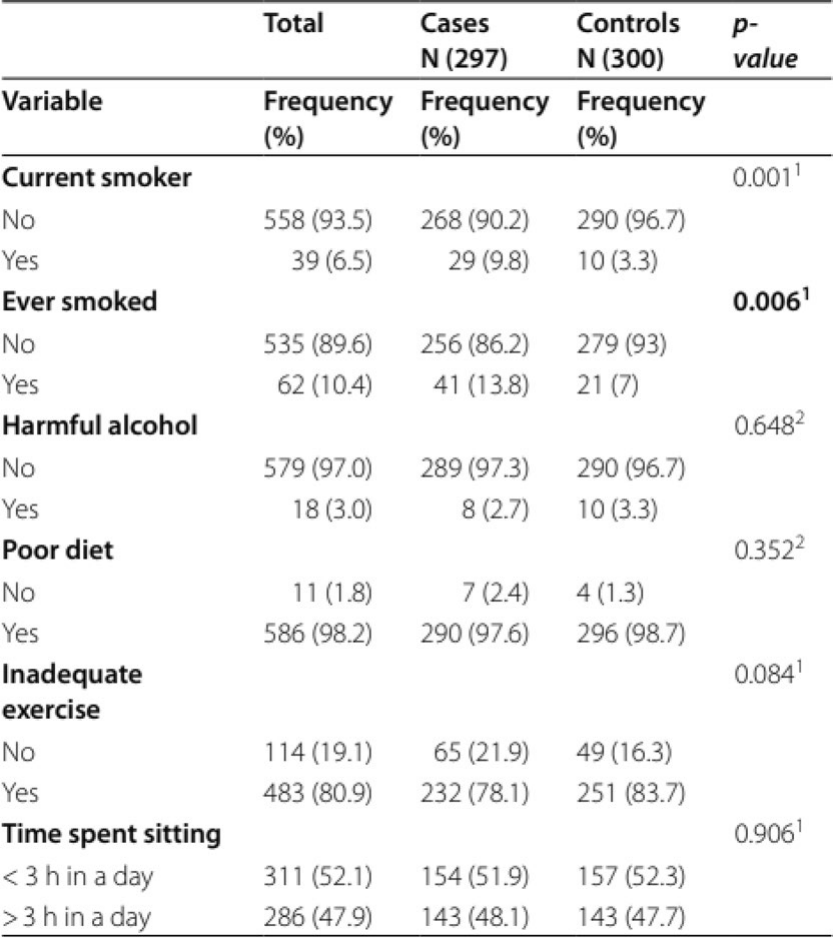


The correct Table 2 is:

**Table 2:** Lifestyle CVD risk factors

Variable	Total	Cases N (297)	Controls N (300)	Χ^2^	*p*-value
	**n (valid %)**	**n (valid%)**	**n (valid %)**		
**Current smoker**				**10.11(1)**	**0.001**^**1**^
No	558 (93.5)	268 (90.2)	290 (96.7)		
Yes	39 (6.5)	29 (9.8)	10 (3.3)		
**Ever smoked**				**7.42(1)**	**0.006**^**1**^
No	535 (89.6)	256 (86.2)	279 (93.0)		
Yes	62 (10.4)	41 (13.8)	21 (7.0)		
**Harmful alcohol**				0.21(1)	0.648^2^
No	579 (97.0)	289 (97.3)	290 (96.7)		
Yes	18 (3.0)	8 (2.7)	10 (3.3)		
**Poor diet**				0.87(1)	0.352^2^
No	11 (1.8)	7 (2.4)	4 (1.3)		
Yes	586 (98.2)	290 (97.6)	296 (98.7)		
**Inadequate exercise**				2.98(1)	0.084^1^
No	114 (19.1)	65 (21.9)	49 (16.3)		
Yes	483 (80.9)	232 (78.1)	251 (83.7)		
**Time spent sitting**				0.01(1)	0.906^1^
< 3 h in a day	311 (52.1)	154 (51.9)	157 (52.3)		
> 3 h in a day	286 (47.9)	143 (48.1)	143 (47.7)		

^1^Chi square test

^2^Fishers’ exact test

The incorrect Table 3 is: 
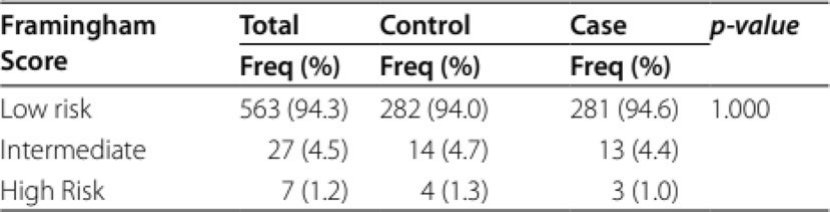


The correct Table 3 is:

**Table 3:** A comparison of Framingham Risk Score between patients and controls

Framingham score	Total n (valid%)	Control n (valid%)	Case n (valid%)	χ^2^	*p*-value
				0.02(2)	1.000
Low risk	563 (94.3)	282 (94.0)	281 (94.6)		
Intermediate	27 (4.5)	14 (4.7)	13 (4.4)		
High Risk	7 (1.2)	4 (1.3)	3 (1.0)		

The incorrect Table 4 is: 
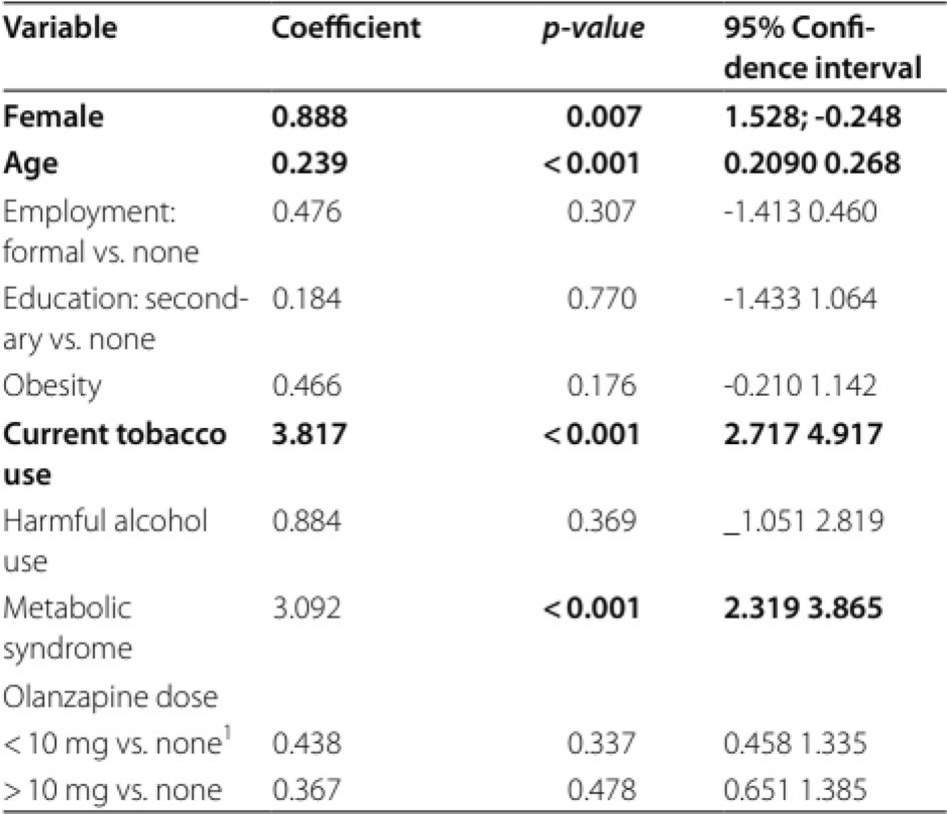


The correct Table 4 is:

**Table 4:** Linear regression model with 10-year Framingham Risk Score as the outcome among patients

	10-year Framingham Risk score			
	Coefficient	SE	T	*p*-value
Female	-1.13	0.35	-3.25	**0.001**
Age	0.24	0.02	14.73	** < 0.001**
Employment				
Formal vs None	-0.52	0.48	-1.09	0.278
Self-employment vs None	-0.58	0.36	-1.61	0.109
Education				
Primary vs None	-0.26	0.62	-0.42	0.673
Secondary vs None	-0.34	0.63	-0.54	0.590
Tertiary vs None	-0.00	0.65	-0.00	0.997
Obesity	0.49	0.34	1.42	0.156
Current tobacco use	3.75	0.57	6.62	** < 0.001**
Harmful alcohol use	1.02	1.00	1.02	0.308
Metabolic syndrome	2.89	0.42	6.88	** < 0.001**
Olanzapine dose				
< 10 mg vs none^a^	0.49	0.47	1.05	0.295
> 10 mg vs none	0.36	0.53	0.67	0.504

^a^Olanzapine was used because 85% of the patients were on olanzapine being a donated drug in this setting

The original article [1] has been corrected.
